# Histidine provides long-term neuroprotection after cerebral ischemia through promoting astrocyte migration

**DOI:** 10.1038/srep15356

**Published:** 2015-10-20

**Authors:** Ru-jia Liao, Lei Jiang, Rong-rong Wang, Hua-wei Zhao, Ying Chen, Ya Li, Lu Wang, Li-Yong Jie, Yu-dong Zhou, Xiang-nan Zhang, Zhong Chen, Wei-wei Hu

**Affiliations:** 1 Department of Pharmacology, Key Laboratory of Medical Neurobiology of the Ministry of Health of China, School of Basic Medical Sciences, School of Medicine, Zhejiang University, Hangzhou, 310058, China; 2 Department of Pharmacology, Collaborative Innovation Center for Diagnosis and Treatment of Infectious Diseases, the First Affiliated Hospital, School of Medicine, Zhejiang University, Hangzhou 310003, China; 3 Department of Pharmacology, Children’s Hospital of Zhejiang University, Hangzhou 310006, China; 4 Department of Radiology, the Second Affiliated Hospital, School of Medicine, Zhejiang University, Hangzhou 310009, China

## Abstract

The formation of glial scar impedes the neurogenesis and neural functional recovery following cerebral ischemia. Histamine showed neuroprotection at early stage after cerebral ischemia, however, its long-term effect, especially on glial scar formation, hasn’t been characterized. With various administration regimens constructed for histidine, a precursor of histamine, we found that histidine treatment at a high dose at early stage and a low dose at late stage demonstrated the most remarkable long-term neuroprotection with decreased infarct volume and improved neurological function. Notably, this treatment regimen also robustly reduced the glial scar area and facilitated the astrocyte migration towards the infarct core. In wound-healing assay and transwell test, histamine significantly promoted astrocyte migration. H2 receptor antagonists reversed the promotion of astrocyte migration and the neuroprotection provided by histidine. Moreover, histamine upregulated the GTP-bound small GTPase Rac1, while a Rac1 inhibitor, NSC23766, abrogated the neuroprotection of histidine and its promotion of astrocyte migration. Our data indicated that a dose/stage-dependent histidine treatment, mediated by H2 receptor, promoted astrocyte migration towards the infarct core, which benefited long-term post-cerebral ischemia neurological recovery. Therefore, targeting histaminergic system may be an effective therapeutic strategy for long-term cerebral ischemia injury through its actions on astrocytes.

Cerebral ischemia is the leading cause of death and disability in adults. Currently, the only approved treatment for cerebral ischemia is recombinant tissue plasminogen activator, which confers reperfusion to the ischemic area when administered within 4.5 h after ischemia. No effective therapeutic neuroprotective agent is yet available for the long-term brain injuries after ischemia. The complex pathophysiological events after cerebral ischemia evolve both temporally and spatially^1^, which hinder the development of a viable neuroprotective agent^2^. For example, NMDA antagonists are effective to alleviate the neuronal excitotoxicity at the early stage after ischemia, while they may not favor the long-term neurological recovery, for NMDA receptors are crucial in neuroplasticity^3^,^4^.

Astrocytes are critically involved in neuronal pathophysiological progresses following cerebral ischemia: as early as 6 h after onset of cerebral ischemia, astrocytes activates to facilitate the survival of neurons possibly *via* antioxidant defense, metabolic support and secretion of neuroprotective substances; those reactive astrocytes also form a barrier to confine the spread of the lesion and the local immune response^5^,^6^. So, an improvement of astrocyte survival is a crucial mean to protect brain against cerebral ischemia^7^. However, the glial scar, a barrier largely composed of astrocytes, may impede the neurogenesis at the late stage of ischemic neuronal injuries. Indeed, the suppression of glial scar may benefit the neurogenesis and neurological recovery^8^. It raises the possibility that dose and stage dependent treatment strategy may be a reasonable way of therapy for ischemic brain injuries by coordinating those time dependent effects of astrocytes.

In our previous study, histamine was found to significantly protect astrocytes from oxygen-glucose deprivation-induced injuries^9^. The astrocytic H1-receptor-mediated up-regulation of glutamine synthetase and glutamate transporter 1 expressions contribute to the protective effect of histamine through the clearance of the redundant extracellular glutamate, which helps to alleviate the excitotoxicity at early stage of cerebral ischemia^9^,^10^. Moreover, considerable evidence shows that histamine provides neuroprotection at early stage after cerebral ischemia^11^,^12^. Intraperitoneal administration of histidine, a precursor of histamine, immediately and 6 h after reperfusion, remarkably alleviates the infarction induced by transient middle cerebral artery occlusion (tMCAO)^11^. Histamine is considered to have a direct neuroprotective effect by alleviating the NMDA-induced excitotoxicity via H2 receptors and cAMP/PKA pathway^13^. Also, enhancement of central histaminergic activity suppresses inflammatory cell recruitment after cerebral ischemia^14^. Taken together, histaminergic system appears to be a potential therapeutic target for cerebral ischemia-induced brain injuries.

However, the long-term effect of histamine after cerebral ischemia has not been investigated, especially its effect on astrocytes at the late stage regarding the glial scar formation. Since histamine cannot penetrate the blood-brain barrier directly, histidine was used to test the long-term effects of histamine on behavioral and histological responses to cerebral ischemia and the potential mechanisms under different stage-related administration regimens.

## Results

### Histidine provides remarkable long-term protection on neurological function and reduces glial scar area after cerebral ischemia

**Figure 1:**
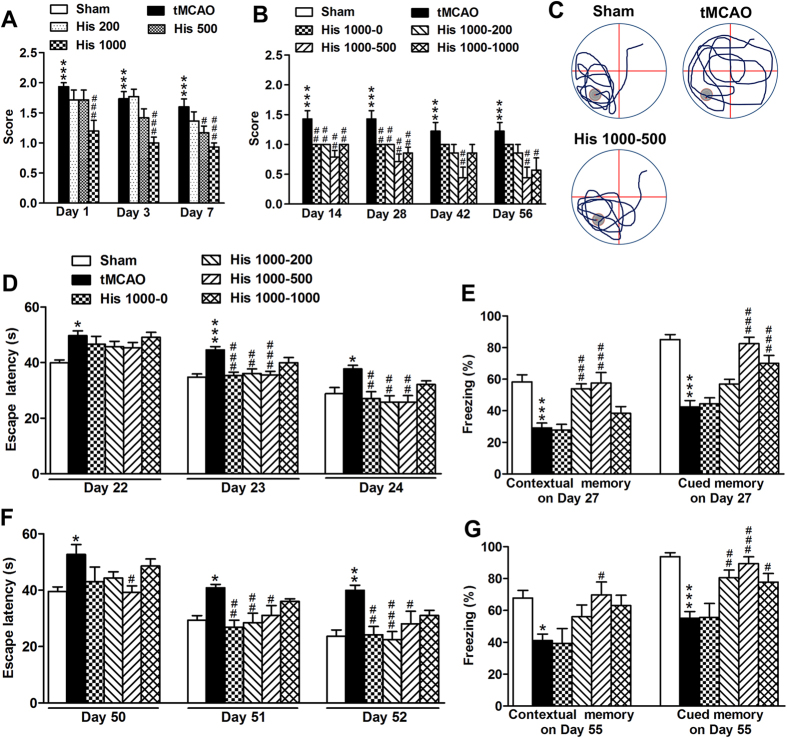
Histidine provides neuroprotection on the neurological function and cognitive abilities after focal cerebral ischemia. Neurological scores were evaluated on 1 d, 3 d, 7 d after ischemia under the treatment of histamine (**A**, 200, 500 or 1000 mg/kg), and evaluated on 14 d, 28 d, 42 d, 56 d after ischemia under the treatment of histidine treatment (**B**, 1000 mg/kg for the first week but 0, 200, 500, or 1000 mg/kg for later weeks, named as His 1000–0, His 1000–200, His 1000–500 or His 1000–1000). Cognitive abilities were examined by Morris water maze (**D**: Day 22–24; **F**: Day 50–52) and contextual and cued fear conditioning test (**E**: Day 27, G: Day 55) after ischemia. The representative swim paths on Day 24 in (**D**) were shown in (**C**), and the gray circle indicates the location of the platform. n = 13–15 for **A**,**B** (Day 14 and Day 28), **D** and **E**; n = 7–9 for **B** (Day 42 and Day 56), **F** and **G**. *P < 0.05, **P < 0.01, ***P < 0.001, compared with sham group within each test day or each test, *^#^P* < 0.05, *^##^P* < 0.01, *^###^P* < 0.001, compared with tMCAO group within each test day or each test.

The pathophysiological events following cerebral ischemia are complicated, *i.e.*, the acute excitotoxicity and inflammatory infiltration usually take place within one week after the onset of ischemia, whereas the glial scar formation and neurogenesis often appears after that^1^. Thus, at first we experimented the doses of histidine (200, 500, or 1000 mg/kg) during the first week, which are often selected dosages in the study of cerebral ischemia^11^,^15^,^16^. We found that the highest dose provided the most prominent protection as evidenced by the neurological deficit score (Fig. 1A; n = 13–15) and measurement of infarct area by MRI (Supplement Fig. 1; n = 5–6). Thus, 1000 mg/kg was chosen as the dosage for the first week, and different other doses were administered for the later weeks. Under different treatment regimens (as indicated as early dosage-late dosage combination), we evaluated the long term effects of histidine on neurological performance and cognitive abilities by using Morris water maze and fear conditioning test, for the memory related brain regions such as striatum, neocortex and amygdala are often compromised after tMCAO^17^,^18^,^19^,^20^. We found that the histidine treatment (1000–500 mg/kg) showed the most remarkable protective effect on the neurological performance (Fig. 1B; n = 13–15 for Day 14 and Day 28; n = 7–9 for Day 42 and Day 56). In Morris water maze test performed from 22 d or 50 d after ischemia, all histidine treatments significantly reduced escape latency in the spatial learning process (analyzed by a general linear model, *P* < 0.05; Fig. 1D: n = 13–15; Fig. 1F: n = 7–9). However, there is no difference among them in probe trial, which refers to the memory retention. In fear conditioning test, Histidine (His) 1000–500 group showed the best contextual memory and cued memory on 27 d and 55 d, compared with other treatment combinations (Fig. 1E: n = 13–15; Fig. 1G: n = 7–9).

**Figure 2:**
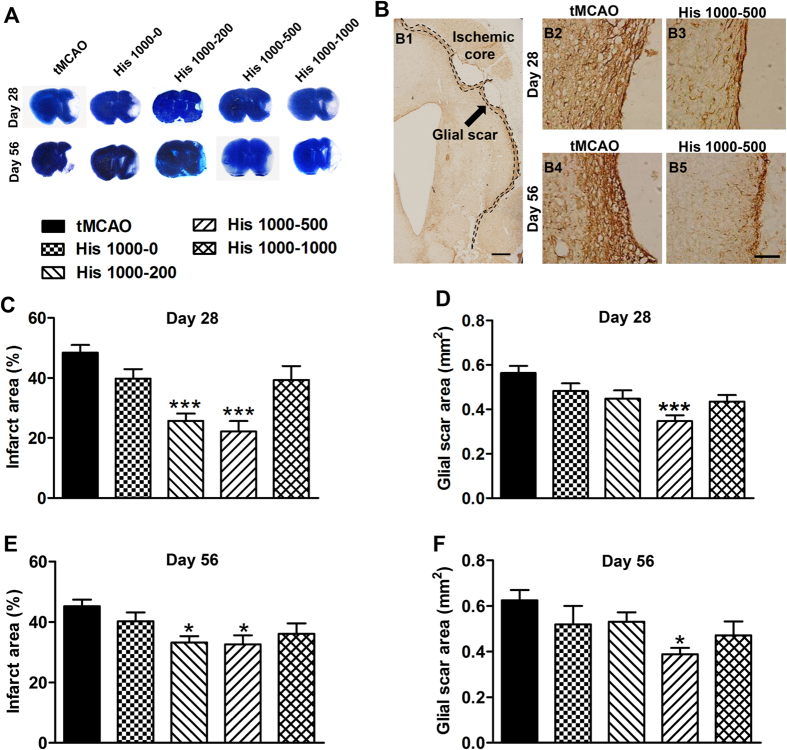
Histidine reduces infarct area and glial scar area after focal cerebral ischemia. Under the different regimens of histidine treatment, the infarct area was estimated with TB staining on 28 d (**C**) and 56 d (**E**) after tMCAO, with the representative layer shown in (**A**). The glial scar area was also estimated with GFAP staining on 28 d (**D**) and 56 d (**F**) after tMCAO, with the representative photo shown in (**B**) (B1 shows the area quantified; B2-B5 shows the enlarged glial scar edge). n = 10–12 for **C** and **D**; n = 6–7 for E and F. B1: bar = 500 μm; B2–5: bar = 100 μm. *P < 0.05, ***P < 0.001, compared with tMCAO group.

We then performed the histological evaluation following different regimens of histidine treatment. By using Toluidine blue (TB) staining on 28 d and 56 d after tMCAO, we found that only the His 1000–200 and 1000–500 combinations remarkably reduced the infarct area (Fig. 2A,C: n = 10–12; Fig. 2E: n = 6–7). The glial scar area around the infarct core was quantified from the GFAP diaminobenzidine histochemistry staining. Only the treatment with His 1000–500 robustly reduced the glial scar area on 28 d and 56 d after ischemia (Fig. 2B,D: n = 10–12; Fig. 2F: n = 6–7). No statistically significant change of glial scar area was detectable between the tMCAO and other histidine treatment groups. Taken together, these data demonstrate that histidine provides long-term neuroprotection after cerebral ischemia, and His 1000–500 treatment displayed the most robust protection on neurological function and reduction of glial scar formation.

### Histidine promotes astrocyte migration towards the infarct core after cerebral ischemia

The glial scar formation usually results from morphological and functional changes of astrocytes that include the activation with GFAP upregulation, proliferation, as well as migration to the edge of lesion^21^,^22^, thus the reduction of glial scar area by histidine may possibly be related to these aspects. To test this hypothesis, astrocytes activation at the penumbra area was examined by the immunohistochemistry and Western blot. After cerebral ischemia, astrocytes were strikingly activated with increased expression of GFAP, however the treatment of His 1000 had no further effect on the activation of astrocytes on 7 d after ischemia, nor did His 1000–0 and 1000–500 treatments on 14 d after ischemia (Supplement Fig. 2; n = 6–7). Subsequently, the proliferation of astrocytes was evaluated based on the quantification of BrdU+/GFAP+ cells at the penumbra area. Although the number of BrdU+ cells increased after the His 1000–500 treatment, the BrdU+/GFAP+ cell numbers was unchanged (Supplement Fig. 3; n = 6–7), which indicates histidine has no effect on the proliferation of astrocytes. The wound healing assay in cultured astrocytes allows us to analyze the direct action of histamine on activation, proliferation and migration of astrocytes. The GFAP expression and the number of BrdU+ cells were unchanged at the wound boundary after the histamine treatment (Supplement Fig. 4). Therefore, the regulation of activation and proliferation of astrocytes unlikely contributed to the reduction of glial scar area by histidine.

**Figure 3:**
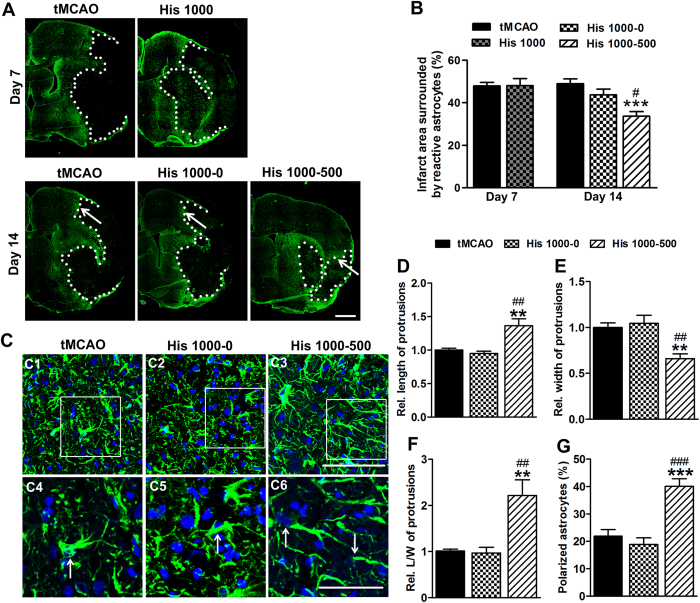
Histidine promotes reactive astrocytes migration towards the infarct core. GFAP immunostaining was performed on 7 d and 14 d after tMCAO (**A**), and the infarct area surrounded by reactive astrocytes was quantified (**B**). The morphology of astrocytes from glial scar edge indicated by arrows in A were shown in C1–3, with enlarged images in C4–6 (GFAP: green; DAPI: blue). Arrows in C4–6 indicate the polarized astrocytes. The length (**D**), the width (**E**), the ratio of length to width of protrusions (**F**) and the percentage of polarized astrocyte (**G**) were quantified at glial scar edge on 14 d after tMCAO. n = 6–8. A: bar = 1 mm; C1–3: bar = 100 μm; C4–6: bar = 50 μm. *P < 0.05, **P < 0.01, ***P < 0.001, compared with tMCAO group, ^#^*P* < 0.05, *^##^P* < 0.01, *^###^P* < 0.001, compared with His 1000–0 group.

To investigate whether the action of histidine on astrocyte migration attributes to the reduction of glial scar area, the distribution of astrocytes was quantified by measuring the infarct area surrounded by reactive astrocytes. On 7 d after ischemia, there was no difference in size of infarct area between control and His 1000 groups, while on 14 d after ischemia the infarct area was notably reduced in His 1000–500 group compared with controls (33.7 ± 2.1% *vs.* 49.0 ± 2.3%, *P* < 0.001; Fig. 3A,B; n = 6–8), which suggests that histidine facilitates astrocytes to migrate towards the infarct core at the late stage of treatment after cerebral ischemia. The fact that infarct area on 14 d was unchanged in His 1000–0 group compared with controls suggests that the treatment with histidine at the late stage was indispensable for the migration of astrocytes towards the infarct core (43.7 ± 2.7% *vs.* 49.0 ± 2.3%; Fig. 3A,B; n = 6–8), which could result in a thinner scar barrier scar formation later on (Fig. 2B,D,F).

Cell migration is a highly orchestrated multistep process. To migrate, a cell first acquires a characteristic polarized morphology in response to extracellular signals, characterized by elongated protrusion^19^. We analyzed the morphology of astrocytes at the glial scar edge (Fig. 3C–G; n = 6–8), and found that the His 1000–500 but not His 1000–0 treatment significantly increased the relative length of (1.36 ± 0.10 *vs.* 1.00 ± 0.03; *P* < 0.01), but reduced the relative width of astrocyte protrusions (0.66 ± 0.05 *vs.* 1.00 ± 0.05; *P* < 0.01), and thus elevated the ratio of length to width (2.22 ± 0.34 *vs.* 1.00 ± 0.04; *P* < 0.01). Additionally, the percentage of polarized cells with the criterion that the length of the protrusion exceeded the width by at least four times, increased in His 1000–500 treatment group (40.1 ± 2.7 *vs.* 21.9 ± 2.4; *P* < 0.001).

### Histamine promotes astrocyte migration *in vitro*

**Figure 4:**
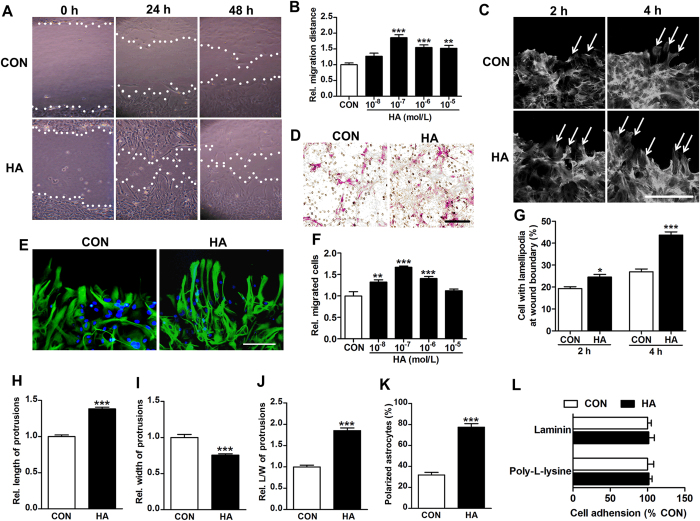
Histamine promotes astrocyte migration in wound-healing assay and transwell migration assay *in vitro*. Phase-contrast micrographs show astrocyte migration under the treatment of histamine (HA) 10^−7^ mol/L at 0 h, 24 h and 48 h after the scratch (**A**, white lines indicate wound edges). The migration distance was quantified under the different concentrations of histamine at 24 h after scratch (**B**). The effect of histamine on astrocyte migration was also analyzed in transwell migration assay (**F**), with representative images of migrated astrocytes under the treatment of histamine 10^−7^ mol/L (**D**). Cells with lamellipodia at wound boundary were counted at 2 h or 4 h after scratch under the treatment of 10^−7^ mol/L histamine (**G**), with representative images stained with rhodamine-phalloidin to show F-actin (**C**, arrows indicate cells with lamellipodia). At 24 h after scratch, the cells under the treatment of histamine 10^−7^ mol/L were stained with GFAP in green and DAPI in blue. The morphology of astrocytes at the wound boundary was analyzed, including the length (**H**), the width (**I**), the ratio of length to width of protrusions (**J**) and the percentage polarized astrocyte (**K**), with the representative images shown in (**E**). The percentage of astrocytes adhering to poly-L-lysine and laminin 30 min after plating was quantified in L. Values are from 3 to 4 independent experiments. C: bar = 50 μm; D, E: bar = 100 μm. *P < 0.05, **P < 0.01,***P < 0.001, compared with control (CON) group.

To confirm the effect of histamine on astrocyte migration, the migration distance was assessed by wound healing assay (Fig. 4A,B; from 3–4 independent experiments). Histamine greatly boosted the migration of astrocytes at the wound boundary, with the dose of 10^−7^ mol/L showing the maximal effect (1.86 ± 0.10 *vs*. 1.00 ± 0.06; *P* < 0.001). In transwell migration assay that is another common test for cell migratory response, again, we found there were more migrated cells after the administration of histamine (Fig. 4D,F; from 3–4 independent experiments). The 10^−7^ mol/L histamine displayed the maximal promotion on astrocyte migration (1.67 ± 0.03 *vs*. 1.00 ± 0.10; *P* < 0.001), whereas such effect was reduced with the dose increasing.

At the cell front, actin assembly drives the extension of flat membrane protrusions called lamellipodia, which contributes to the cell polarization for migration^23^. As shown by a staining of filamentous actin (F-actin), histamine increased the percentage of cells with lamellipodia at wound boundary (Fig. 4C,G). We then examined the morphology of astrocytes at wound boundary by GFAP immunostaining. As shown in Fig. 4E,H–K (from 3–4 independent experiments), similar to that *in vivo*, histamine remarkably increased the relative length of protrusions (1.38 ± 0.02 *vs*. 1.00 ± 0.02; *P* < 0.001), ratio of length to width of protrusions (1.85 ± 0.06 *vs*. 1.00 ± 0.04; *P* < 0.001), percentage of polarized cells (77.5 ± 3.3 *vs*. 31.9 ± 2.5; *P* < 0.001), but reduced the relative width of protrusions (0.75 ± 0.02 *vs*. 1.00 ± 0.04; *P* < 0.001). Following the polarization, cells form adhesions that connect the extracellular matrix to the actin cytoskeleton to anchor the protrusion and tract the cell body, therefore the alteration of adhesion could also influence the migration^24^. However, our study indicated that the adhesion ability of astrocytes was unchanged after histamine treatment, which was tested on either poly-L-lysine or laminin coated surface (Fig. 4L). Together, these results suggest that histamine facilitate the migration of astrocytes towards the infarct core probably through promoting astrocyte polarization.

### Histidine’s neuroprotection effect and its astrocyte migration promotion are mediated through H2 receptor

**Figure 5:**
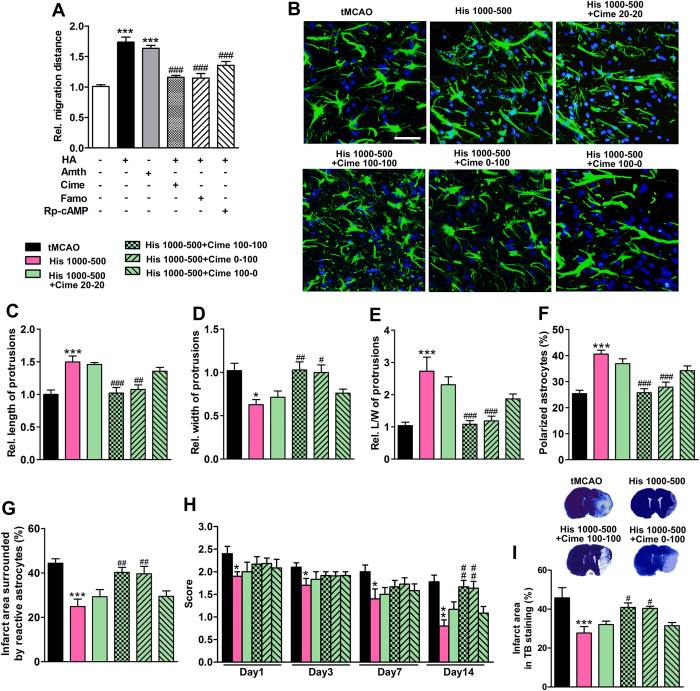
Histidine promotes astrocyte migration and provides neuroprotection through H2 receptor. In wound-healing assay, cimetidine (Cime) at 10^−7^ mol/L, famotidine (Famo) at 10^−7^ mol/L, or Rp-cAMP at 10^−5^ mol/L was administrated after the scratch, while 10^−7^ mol/L histamine (HA) or 10^−8^ mol/L amthamine (Amth) was added 30 min later. Their effects on astrocyte migration were shown in (**A**). Cimetidine treatments were also divided into two phases (first week and later week), during which cimetidine was not given or given at the dose of 20 or 100 mg/kg at 30 min before each injection of histidine, including Cime 20–20, Cime 100–100, Cime 0–100 and Cime 100–0 regimens. The morphology of astrocytes from the glial scar edge 14 days after tMCAO was shown in B (GFAP: green; DAPI: blue). The length (**C**), the width (**D**), the ratio of length to width of protrusions (**E**) and the percentage polarizing astrocyte (**F**) were quantified. The infarct area surrounded by reactive astrocytes was quantified in (**G**). The neurological deficit score was evaluated on 1 d, 3 d, 7 d and 14 d after tMCAO (**H**), while the infarct area was also estimated with TB staining (**I**). A: values are from 3 to 4 independent experiments; **B**–**I**: n = 10–12. Bar = 50 μm. *P < 0.05, **P < 0.01, ***P < 0.001, compared with control group (**A**) or tMCAO group (**C**–**I**) within each test day or each test; ^#^*P* < 0.05, ^##^*P* < 0.01, ^###^*P* < 0.001, compared with histamine or His 1000–500 group within each test day or each test.

Both histamine H1 and H2 receptors have been found in astrocytes^25^,^26^, whereas their exact functions are largely unknown. We found H2 agonist amthamine had similar action as histamine on the migration of astrocyte in wound healing assay, while H2 antagonist cimetidine and famotidine both abrogated the promotional effect of histamine on astrocyte migration (Fig. 5A; from 3–4 independent experiments). On the other hand, H1 antagonist pyrilamine cannot inhibit the aforementioned action of histamine (Supplement Fig. 5). PKA is in the downstream signal pathway of histamine H2 receptor activation^27^. We found PKA inhibitor Rp-cAMP also reversed the histamine-promoted astrocyte migration (Fig. 5A; from 3–4 independent experiments), which further confirmed the involvement of H2 receptor in the action of histamine on astrocyte migration. Moreover, administration of cimetidine, famotidine, pyrilamine, and Rp-cAMP alone had no effect on the migration of astrocytes.

To verify the involvement of H2 receptor in the actions of histidine *in vivo*, cimetidine was injected before each histidine treatment (Fig. 5B–I). We found that the polarization of astrocytes at the glial scar edge was also blunted by cimetidine (100 mg/kg) injection during 0–14 d or 7–14 d (referred to Cime 100–100 or Cime 0–100 combinations, respectively), which was manifested by the shortened length of protrusions and reduced percentage of polarized cells but increased protrusion width, compared with the treatment with His 1000–500 alone (Fig. 5B–F; n = 10–12). The reduction of infarct area surrounded by reactive astrocytes after the His 1000–500 treatment was also abrogated by cimetidine (100 mg/kg) injection during 0–14 d or 7–14 d after ischemia (Fig. 5G; n = 10–12). However, the treatment of cimetidine during 0–7 d (100 mg/kg, referred to Cime 100–0 combination treatment) had no such effect, suggesting that blocking H2 receptor, especially at the late stage, reversed the astrocyte migration promotion by histidine.

The neurological deficit score and infarct area were also evaluated after the cimetidine injection (Fig. 5H, I; n = 10–12). Again, cimetidine (100 mg/kg) injection during 0–14 d or 7–14 d, but not 0–7 d reversed the histidine-induced reduction of neurological deficit score assessed on 14 d after cerebral ischemia, when the migration of astrocytes has also occurred (Fig. 5H; n = 10–12). In parallel, the reduction of infarct area provided by histidine was abrogated by cimetidine (100 mg/kg) injected during 0–14 d or 7–14 d (Fig. 5I; n = 10–12). On the other hand, H1 receptor antagonist pyrilamine has no effect on the action of histidine on astrocyte polarization (Supplement Fig. 5). These data indicate that the promotional effect of histamine on astrocyte migration is mediated by H2 receptor, which may contribute to its neuroprotective effect.

### The histidine-induced improved recovery after cerebral ischemia is reversed by Rac1 inhibitor through the blockade of astrocyte migration

**Figure 6:**
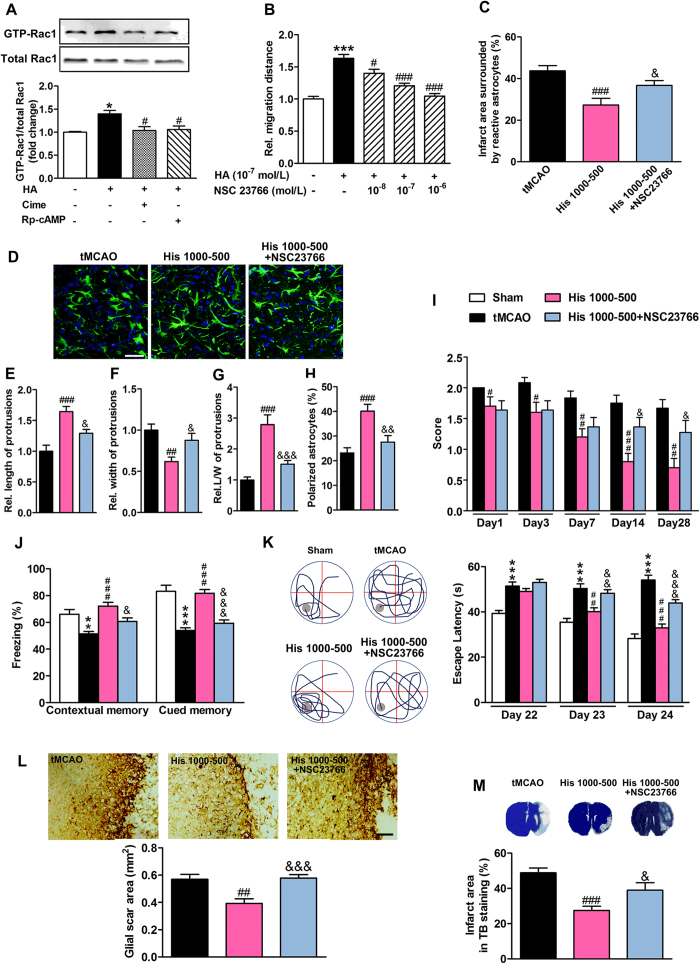
Inhibition of small GTPase Rac1 impedes astrocyte migration and neurological recovery provided by histidine. In wound-healing assay, cimetidine (Cime, 10^−7^ mol/L) or Rp-cAMP (10^−5^ mol/L), Rac1 inhibitor NSC 23766 at indicated concentration were administrated after the scratch, while 10^−7^ mol/L histamine (HA) was added 30 min later. The GTP-bound small GTPase Rac1 was examined by Western blotting analysis at 24 h after scratch (**A**). The quantification of migration distance at 24 h after scratch was shown in B. After tMCAO, 50 μg NSC23766 was delivered into cerebral ventricle at 30 min before each injection of histidine from 7 d to 14 d after tMCAO. Its effects on the infarct area surrounded by reactive astrocytes and on the morphology of astrocytes at the glial scar edge 14 days after tMCAO were shown in (**C**,**D**) with GFAP immunostaining (GFAP: green; DAPI: blue). The length (**E**), the width (**F**), the ratio of length to width of protrusions (**G**) and the percentage polarized astrocyte (**H**) were quantified. The neurological deficit score (**I**) was evaluated on 1 d, 3 d, 7 d, 14 d and 28 d after tMCAO, while the cognitive abilities were evaluated by contextual and cued fear conditioning test (Day 27, **J**) and Morris water maze (Day 22–24, **K**) with representative swim paths (Day 24, the gray circle indicates the location of the platform). The glial scar area (**L**) and infarct area (**M**) from TB staining were determined on 28 d after tMCAO. **A** and **B**: values are from 3 to 4 independent experiments; **C**–**L**: n = 10–12. **D**: bar = 50 μm; **L**: bar = 100 μm. *P < 0.05, **P < 0.01, ***P < 0.001, compared with control group (**A**,**B**) or sham group (**J**,**K**) within each test day or each test; *^#^P* < 0.05, *^##^P* < 0.01, *^###^P* < 0.001, compared with histamine treatment group **A** and **B**) or tMCAO group (**C**–**M**); &*P* < 0.05, &&*P* < 0.01, &&&*P* < 0.001, compared with His 1000–500 group within each test day or each test.

Numerous studies have revealed that Rho GTPases, including RhoA, Rac1 and CDC42 are crucial for the signaling pathways underlying the establishment of polarization that precedes cell migration, among which Rac1 is thought to be the major activator in the formation of lamellipodia^23^. We found that histamine increased the level of active Rac1 as examined by GTPase pull-down assay that tests GTP-bound Rac1, while cimetidine and Rp-cAMP both abolished the up-regulation of Rac1 by histamine (Fig. 6A; from 3–4 independent experiments). Moreover, Rac1 inhibitor NSC23766 reversed the histamine-induced promotion of astrocyte migration (Fig. 6B; from 3–4 independent experiments), which suggests that histamine may facilitate astrocyte migration through H2 receptor and the subsequent up-regulation of active Rac1.

To further verify the involvement of astrocyte migration in the protective effect of histidine, Rac1 inhibitor NSC23766 was delivered into the cerebral ventricle during 7–14 d after tMCAO, during which time the astrocytes migrated towards the infarct core (Fig. 3A,B). The infarct area surrounded by reactive astrocytes was enlarged after the delivery of NSC23766 along with histidine treatment (Fig. 6C; n = 10–12). The polarization of astrocytes was also abrogated by NSC23766, as demonstrated by the increases in the length of, the ratio of length to width of the protrusions, and percentage of polarizing cells, but the decrease in the width of protrusion (Fig. 6D–H; n = 10–12), which suggests that NSC23766 can inhibit histidine-upregulated astrocyte migration. NSC23766 also reversed the histidine-induced improvement of neurological function after cerebral ischemia (Fig. 6I; n = 10–12). In the fear conditioning test and Morris water maze test, NSC23766 robustly reversed the histidine-conferred improvement of cognitive abilities (Fig. 6J,K; n = 10–12). Furthermore, NSC23766 abrogated the histidine-induced reduction of glial scar area (Fig. 6L; n = 10–12; 0.58 ± 0.03 *vs*. 0.39 ± 0.03; *P* < 0.001) and the infarct area as indicated by TB staining (Fig. 6M; n = 10–12; 38.9 ± 4.2 *vs*. 27.5 ± 2.4; *P* < 0.05). Together these findings suggest the migration of astrocytes is crucial for the neuroprotection effect of histidine after cerebral ischemia.

## Discussion

Histamine has been reported to offer neuroprotection at early stage after cerebral ischemia, which is attributed to its action on neurons, astrocytes or inflammatory cells^28^. Histidine and its precursor carnosine also have direct neuroprotection at very early stage after the onset of cerebral ischemia with their antioxidant and anti-apoptotic properties^29^,^30^. However, the action of histamine or its related agents at late stage after cerebral ischemia hasn’t been investigated. Here, through behavioral and pathological evaluations, we found that histidine, which is the precursor of histamine, provided long-term neuroprotection in a dose and stage dependent manner. This protection may be largely due to the restriction of glial scar formation through promoting astrocyte migration. Our study highlights the regulation of astrocyte function as a therapeutic strategy for cerebral ischemia-induced brain injuries.

The dose and stage dependent regimen of histidine treatment was proposed based on the results of behavioral tests, pathological examination and *in vitro* experiment (Figs 1, 2 and 4), as following: 1) histidine at a consistent dose of 1000 mg/kg has improved neurological function, which is nevertheless not as remarkable as the treatment with dose and stage dependent regimen; 2) histidine (1000 mg/kg) at the early stage only (refers to the His 1000–0 group) has no protective effect on neurological deficit and fear memory at the late stage; 3) histidine at a high dose of 1000 mg/kg at early stage and low dose of 200 or 500 mg/kg at late stage produced superior protection regarding neurological function and infarct area, among which the regimen with dose of 500 mg/kg at late stage has the most prominent protective effect, whereas a regimen with reversed dose sequence with low dose at early stage but high dose at late stage has no protection (data not shown); 4) only the treatment with His 1000–500 reduced the glial scar area; 5) the promotion of astrocyte migration *in vitro* decreases as the dose of histamine increases, suggesting high dose of histamine may not benefit the astrocyte migration that takes place at the late stage. Therefore, our study suggested that the dose and stage dependent treatment strategy is effective for the therapy of cerebral ischemia.

The underlying factors contributing to the protective effect from this dose and stage dependent regimen can be intricate, probably due to the multiple actions involved against cerebral ischemia-induced brain injury. Histidine at a dose of 1000 mg/kg administered at the early stage after onset of ischemia reduce infarct volume and neuronal death, and its actions on neuronal survival, astrocytic glutamate clearance and inhibition of inflammatory cell infiltration are suggested to be the underlying mechanisms^11^,^28^. These acute or sub-acute events usually take place within days or one week after ischemia when the rescue of astrocytes at this stage often performs a beneficial role, whereas astrocytes gradually form a firm barrier to impede neuronal reconstruction after that. Although a strict treatment regimen based on time course hasn’t been investigated, under the present high and low dose combined regimen, histidine remarkably reduced the formation glial scar barrier, which may underlie its prominent long-term protection against behavioral deficiency and infarction. Therefore, histaminergic system likely can be a promising therapeutic target through its orchestrating astrocyte actions after cerebral ischemia in a dose and stage dependent manner.

In addition, our study indicated that histidine or histamine did not affect the proliferation and activation (Supplement Figs 2–4), but only the migration of astrocytes (Fig. 3), which resulted in a thinner glial scar barrier (Figs 2B,D,F and 6L). Furthermore, we found that promotion of astrocyte migration contributes to the protection, evident by the fact that the blockage of astrocyte migration by NSC23766 abolished the histidine-induced neuroprotection (Fig. 6). Other studies also suggested the contribution of astrocyte migration to the neuroprotection. In spinal cord injury, a beneficial effect was observed after the stimulation of migrating astrocytes through glycogen synthase kinase-3 inhibition^31^. Moreover, adrenomedullin provides neuroprotection against cerebral ischemia-induced injury while enhancing astrocyte migration^32^. To be noted, this migration is often referred to a moving or shifting of astrocytes towards the infarct core, which may be different from the migration of astrocyte to form glial scar, in which astrocytes accumulate at the penumbra area.

One direct concern over the benefits from astrocyte migration may be that it leads to a compacted glial scar to form a thinner barrier (Figs 2B,D,F and 6L) and generates a larger penumbra area without glial scar (Figs 3A,B and 6C), so as to subserve the neurogenesis in the penumbra area. This notion is plausible since more newborn neurons were found at the glial scar edge after histidine treatment, which was abrogated by Rac1 inhibitor NSC 23766 along with the blockade of astrocyte migration (Supplement Fig. 6). Also, the migrated astrocytes may constrict the inflammatory cells into the infarct core, which has been confirmed in spinal cord injury^31^. Moreover, it is worth noting that control of glial scar formation *via* manipulating astrocyte migration may be a superior approach to produce neuroprotection, since other ways to achieve less glial scar formation such as inhibition of astrocyte activation and proliferation may affect the normal function of astrocytes and cause the lesion area to spread at the early stage of ischemia. Indeed, ablation of astrocyte activation by using astrocyte GFAP and vimentin double knockout mice enlarged the infarct volume, which may be related to the changes of glutamate transport, gap junctions, and plasminogen activator inhibitor-1 expression in astrocytes^33^.

The astrocyte migration is mediated by H2 receptors, as indicated in our investigation with the application of antagonist and agonist of H2 receptor and through blockage of its signaling pathway *in vitro* (Fig. 5). Moreover, H2 antagonist, but not H1 antagonist, also inhibited astrocyte migration and reversed the histidine-induced reduction of infarct area. To be noted, during the entire course of treatment with H2 antagonist, effects similar to that described above were only observed at the late stage after cerebral ischemia, during which time the astrocyte migration develops. Thus, activation of H2 receptor in astrocytes facilitates the astrocyte migration towards the infarct area conferring neuroprotection at least at the late stage after cerebral ischemia, while H1 receptor is probably not involved (Supplement Fig. 5).

The multitude actions of histamine after cerebral ischemia may largely rely on the participation of its multiple receptors such as H1 and H2 receptors, which affects different cells *via* different actions^12^,^28^,^34^,^35^. Our study revealed a novel effect of histamine on astrocyte migration through H2 receptor to provide long-term neuroprotection. In addition, direct effect of histidine on cognitive impairments after cerebral ischemia cannot be excluded, since histamine has been found to improve the learning and memory^36^,^37^,^38^. Delivery of histamine in hippocampus ameliorates spatial memory deficits in radial arm maze task via both H1 and H2 receptors^36^. Bilateral post-training injections of the H2 receptor agonists amthamine or RAMH into the dorsal hippocampus facilitate memory consolidation after contextual fear-conditioning^37^. So, histamine or its related agents may be potential candidates for the functional recovery during the long-term cerebral ischemia injury.

Rho GTPases play central roles in cell migration, among which are the Rac1 induced lamellipodia formation^38^. A high ratio of polarized astrocytes and lamellipodia formation was observed in cultured astrocytes treated with histamine or in glial scar edge in tissue from histidine-treated rats. Furthermore, the active GTP-bound small GTPase Rac1 was up-regulated by histamine, which can be reversed by cimetidine or Rp-cAMP. NSC 23766, a Rac1-specific inhibitor abrogated the histamine-induced promotion of astrocyte migration. Therefore, histamine promotes astrocyte migration towards the infarct area through H2 receptor and subsequent activation of Rac1, which enriches our knowledge about the role of histamine in astrocyte migration in CNS.

In conclusion, our data indicate that a dose and stage dependent treatment with histidine promotes astrocyte migration towards the infarct area through H2 receptor and subsequent up-regulation of active Rac1, which benefits long-term neurological function recovery after cerebral ischemia. It also suggests that targeting histaminergic system may be a new therapeutic strategy for long-term cerebral ischemia-induced injury by its actions on astrocytes.

## Methods

### Animals

The male Sprague-Dawley (SD) rats weighing 250–280 were used for *in vivo* experiments, and the SD neonatal rats were used for astrocyte cultures experiments. All experiments were approved by and conducted in accordance with the ethical guidelines of the Zhejiang University Animal Experimentation Committee and were in complete compliance with the National Institutes of Health Guide for the Care and Use of Laboratory Animals. Efforts were made to minimize any pain or discomfort, and the minimum number of animals was used. In all following experiments, animals were randomized into control and treatment groups.

### Transient focal cerebral ischemia

Rats were anesthetized by intraperitoneal injection of choral hydrate (350 mg/kg). Transient focal cerebral ischemia was induced by tMCAO, as previously described^39^. Briefly, a 6–0 nylon monofilament suture, blunted at the tip and coated with 1% poly-L-lysine, was advanced 18 mm into the internal carotid to occlude the origin of the middle cerebral artery (MCA). Cerebral blood flow (CBF) in the territory of the middle cerebral artery was determined by laser Doppler flowmetry^40^ (Periflux System 5010; Perimed, Jarfalla, Sweden). A flexible fiber-optic probe was affixed to the skull over the cortex supplied by the proximal part of the right MCA (2 mm caudal to bregma and 6 mm lateral to midline). Animals with  <80% reduction in CBF in the core of the MCA territory were excluded from the study. After 90 min of occlusion, reperfusion was performed by removing monofilament. The rats, whose post- reperfusion CBF does not reach 60% of previous CBF before occlusion, were excluded. Body temperature was maintained at 37 °C by a heat lamp (FHC, Bowdoinham) during surgery and for 2 h after the start of reperfusion.

### Treatment regimens

There were 4 separate experiments. Within each, rats were randomized to treatment group as follows (Supplement Fig. 7). Experiment 1, rats were given 200, 500 or 1000 mg/kg histidine during the first week. Experiment 2, rats were given 1000 mg/kg histidine during the first week but 0, 200, 500 or 1000 mg/kg histidine during later weeks, which are named as His 1000–0, His 1000–200, His 1000–500 or His 1000–1000 groups. For histidine treatment, histidine (Sigma, USA) was dissolved in normal saline and injected intraperitoneally at 0 h, 6 h and every other day after tMCAO. For the controls, rats were injected with the same volume of saline. Experiment 3, along with His 1000–500 treatment, cimetidine treatments were also divided into two phases (first week and later week), during which cimetidine (20 or 100 mg/kg) was given intraperitoneally 30 min before each injection of histidine, including Cime 20–20, Cime 100–100, Cime 0–100 and Cime 100–0 regimens. Experiment 4, along with His 1000–500 treatment, Rac1 inhibitor NSC 23766 (50 μg) was delivered to the contralateral ventricle 30 min before each injection of histidine during the second week after tMCAO. The neurological function were scored at 1, 3, 7, 14, 28, 42, 56 days after tMCAO^39^ and cognitive abilities in Morris water maze (Day 22–25, Day 50–53) and contextual fear conditioning (Day 26–27, Day 54–55) were evaluated before sacrifice^41^,^42^,^43^. Rats were sacrificed at 7, 14, 28 or 56 days after tMCAO for immunohistochemistry, Western blot or TB staining. Inclusion and exclusion animal numbers for each group were shown in Supplementary Table 1.

### Wound healing assay

Primary cortical astrocyte cultures were prepared from 1–2 d postnatal SD rats as described previously^44^. Scratches were made on the cell layer by using sterile 20 μl pipette tip. The plates were then rinsed with sterile PBS to remove cell debris and replaced with fresh cell culture media supplemented with 1% fetal bovine serum (FBS). Cimetidine, Rp-cAMP or NSC23766 at indicated concentration was added into the medium replacement, while histamine was added 30 min later. At 0, 24 and 48 h after scratch, cells were stained with GFAP or observed under phase contrast microscope (Olympus, Japan). The distance between the two edges of the scratch was determined by measuring the wound area divided by the length of the scratch using NIH Image J software. For small GTPases analysis, cell protein samples were prepared at 2 h after scratch.

### Immunohistochemistry and immunocytochemistry

For immunohistochemistry, the rats were anesthetized with choral hydrate (350 mg/kg), and transcardially perfused with ice cold normal saline and 4% paraformaldehyde. Brains were removed and post-fixed in 4% paraformaldehyde at 4 °C for 24 h, and then in 30% sucrose in PBS for 3 d. Frozen brain sections were cut at 10 μm on a cryostat (Leica, Germany). For immunocytochemistry, astrocytes monolayer was fixed in 4% paraformaldehyde for 10 min. The cultured astrocytes or the brain sections were then incubated with 3% normal donkey serum in PBS containing 0.1% or 0.3% Triton X-100 for 15 min, respectively. Then primary antibody for GFAP (1:400, Boster, China) was applied for overnight incubation at 4 °C. After repeated wash in PBS, Alexa 488 conjugated anti-rabbit IgG (1:400; Invitrogen, USA) was applied for 2 h incubation at room temperature. For F-actin staining, cells were incubated in rhodamine-phalloidin (1:500, Invitrogen, USA) for 30 min instead of the antibody incubation. After repeated washes, the sections or the cultured astrocytes were mounted in mounting media containing 4′,6-diamidino-2-phenylindole (DAPI, 1:1000; Sigma, USA). Finally, images were taken under a fluorescence microscope (Olympus BX51; Japan). For glial scar quantification, diaminobenzidine histochemistry staining was performed. Endogenous peroxidases were quenched by treatment with 3% H_2_O_2_ in methanol, and slides were blocked with 10% normal goat serum. Slides were then stained with antibody for GFAP (1:200, Boster, China) and were processed by Histostain-Plus IHC Kit (MR Biotech, China). Then the sections were mounted after being washed three times for 10 min with PBS. The glial scar area measure, cell counting and morphology analysis were performed by Image-Pro Plus (Media Cybernetics, Silver Spring, USA) and Image J software (NIH, Bethesda, MD). The length of protrusions was determined as the distance from back of nucleus to the tip of the protrusions. The width was determined at the bottom of the protrusions. Polarized cells were scored when the length of the protrusion exceeded the width of the protrusion at least four times^45^. Totally about 500 astrocytes from the glial scar area of 3 slices were examined for each animal.

### Quantification of infarct area

To quantify the infarct area, serial coronal brain sections were cut at 30 μm on a cryostat (Leica, Germany) and collected every 1.5 mm throughout the entire brain. The slices were stained with 1% TB to define non-infarct tissue. The percentage of the infarct area was calculated as 100 times the ratio of infarct area to the total contralateral hemispheric area. The infarct area surrounded by reactive astrocytes was also estimated after the GFAP immunostaining by the same means.

### Western blot

The cultured astrocytes were homogenized in protein extraction reagent. The active GTP-bound Rac1 were isolated with GTPase Pull-Down kit (Thermo, USA). Protein samples were then separated on 12.5% SDS-polyacrylamide gels and then electrotransferred onto a nitrocellulose membrane. After blocking with 5% non-fat milk, the membranes were incubated with primary antibodies against rac 1 (1:500; Millipore, USA) and GAPDH (1:3,000; KangChen, China) at 4 °C overnight. After repeated washes, the membranes were reacted with IRDye 800 anti-rabbit Molecular Probe (1:8000, LI-COR Biosciences, USA) or IRDye 700 anti-mouse Molecular Probe (1:3000, LI-COR Biosciences, USA) for 2 h. Images were acquired with the Odyssey infrared imaging system (LI-COR Biosciences, USA) and analyzed.

### Migration assay

Migration assay was performed with BD matrigel^TM^ invasion chamber (24 well) according to previous reports^46^. Briefly, astrocyte suspensions (2 × 10^6^/ml, 0.2 ml containing 1% FBS) were added into the transwell inserts, while histamine at indicated concentration was loaded into the bottom wells containing 5% FBS. After 24 h of incubation, the insert were removed from the transwell and non-migrating cells were removed by gently swiping the interior of the transwell with a cotton swab. The migrated cells were then fixed with 4% paraformaldehyde and stained with crystal violet. For quantitative analysis, the number of migrated cells in each insert was counted.

### Adhesion assay

The astrocytes at 3 × 10^5^/ml were sub-cultured in 10^−7^ mol/L histamine for 30 min on 96-well culture plates, which were previously coated by poly-L-lysine or laminin^38^. After repeated wash with PBS, the cells were incubated with MTT (Sigma, USA, 0.5 mg/mL as final concentration) for 4 h at 37 °C. Then, the supernatant layer was removed, and 100 μL of dimethyl sulfoxide was added to each well. MTT metabolism was quantified spectrophotometrically at 570 nm by a Biotek microplate reader (USA).

### Statistical analysis

All data were collected and analyzed in a blind fashion. Data are presented as mean ± S.E.M. The neurological deficit scores within each test day are analyzed by the nonparametric Kruskal-Wallis H-test. Other behavioral data within each test day or from each test, and other multiple comparisons in pathological examination and *in vitro* cell culture experiments were analyzed by One-way ANOVA followed by *Tukey* test, while two tailed-Student’s *t*-test was applied for other comparisons between two groups. Furthermore, a general linear model was used to analyze the difference in latency among different treatment groups in Morris water maze test, with the consideration of all the test days. For all analyses, the tests were two-sided and a *P*  <  0.05 was considered significant. The sample size calculation is based on the formula: 
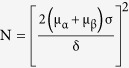
; μ_α_ = 1.62(α = 0.05); μ_β_ = 1.28(β = 0.10); σ = the average S.D.; δ = the average difference among groups.

## Additional Information

**How to cite this article**: Liao, R.-J. *et al.* Histidine provides long-term neuroprotection after cerebral ischemia through promoting astrocyte migration. *Sci. Rep.* **5**, 15356; doi: 10.1038/srep15356 (2015).

## Supplementary Material

Supplementary Information
