# Supplementary material for: Histidine provides long-term neuroprotection after cerebral ischemia through promoting astrocyte migration
Source: Sci Rep. 2015 Oct 20;5:15356. doi: 10.1038/srep15356 (PMC4611873; doi:10.1038/srep15356)
Supplement: Supplementary Information [file srep15356-s1.doc]

***Submitted to Scientific Reports***

**Histidine provides long-term neuroprotection after cerebral ischemia through promoting astrocyte migration**

Ru-jia Liao^a,e^, Lei Jiang^a,e^, Rong-rong Wang^a,b,d^, Hua-wei Zhao^c^, Ying Chen^a^, Ya Li^a^, Lu Wang^a^, Li-Yong Jie^d^, Yu-dong Zhou^a^, Xiang-nan Zhang^a^, Zhong Chen^a,*^, Wei-wei Hu^a,*^

^a^Department of Pharmacology, Key Laboratory of Medical Neurobiology of the Ministry of Health of China, College of Pharmaceutical Sciences, School of Medicine, Zhejiang University, Hangzhou, 310058, China; ^b^Department of Pharmacology, the First Affiliated Hospital, School of Medicine, Zhejiang University, Hangzhou 310003, China; ^c^Department of Pharmacology, Children’s Hospital of Zhejiang University, Hangzhou 310006, China; ^d^Department of Radiology, the Second Affiliated Hospital, School of Medicine, Zhejiang University, Hangzhou 310009, China;

^e^These authors contributed equally to the paper.

*Correspondence to:

Weiwei Hu, Ph.D., Associate Professor, Tel: +86-571-88208227; Fax: +86-571-88208228; mail address: huww@zju.edu.cn.

Zhong Chen, Ph.D., Professor, Tel&Fax: +86-571-88208228; mail address: chenzhong@zju.edu.cn.

**Supplementary Information**

**
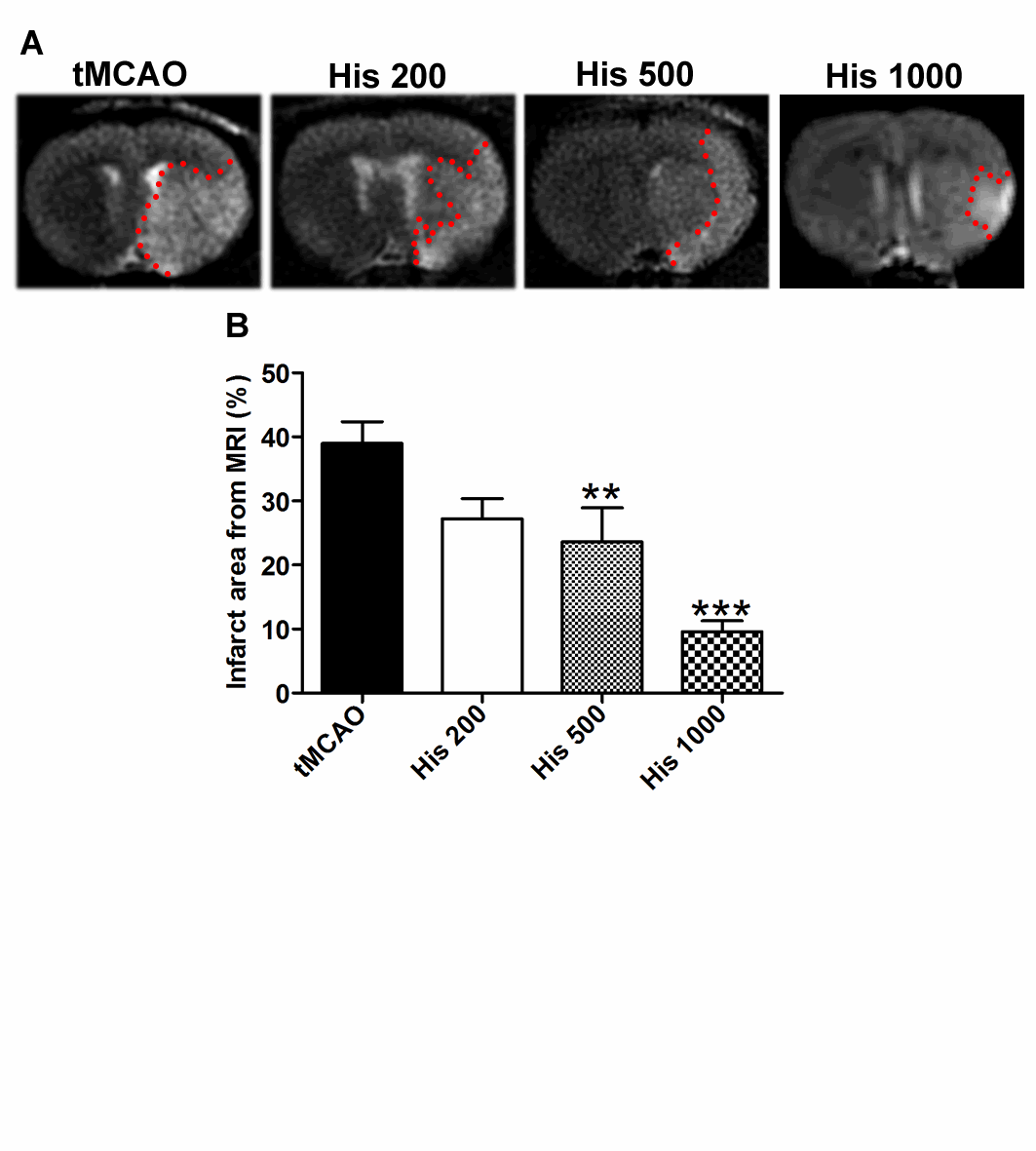
**

**Supplement Fig.1 Histidine reduces infarct area from the magnetic resonance imaging (MRI) examination.** On Day 7 after tMCAO, MRI was performed by 3 Tesla using a Discovery MRI750 scanner (GE, U.S.A.). T2-weighted multi-slice images were acquired using a RARE sequence with the following parameters: time-to-repetition = 3000 ms, effective time-to-echo = 90 ms, matrix size of 320×320, field-of-view FOV of 60 mm × 60 mm, 16 slices with a slice thickness of 2 mm, and layer spacing 0.2 mm. The percentage of the infarct area (B) was calculated as 100 times the ratio of infarct area (outlined by red dot line) to the total contralateral hemispheric area with representative images in A. n=5-6. **P*<0.05, ****P*<0.001, compared with tMCAO group.

**
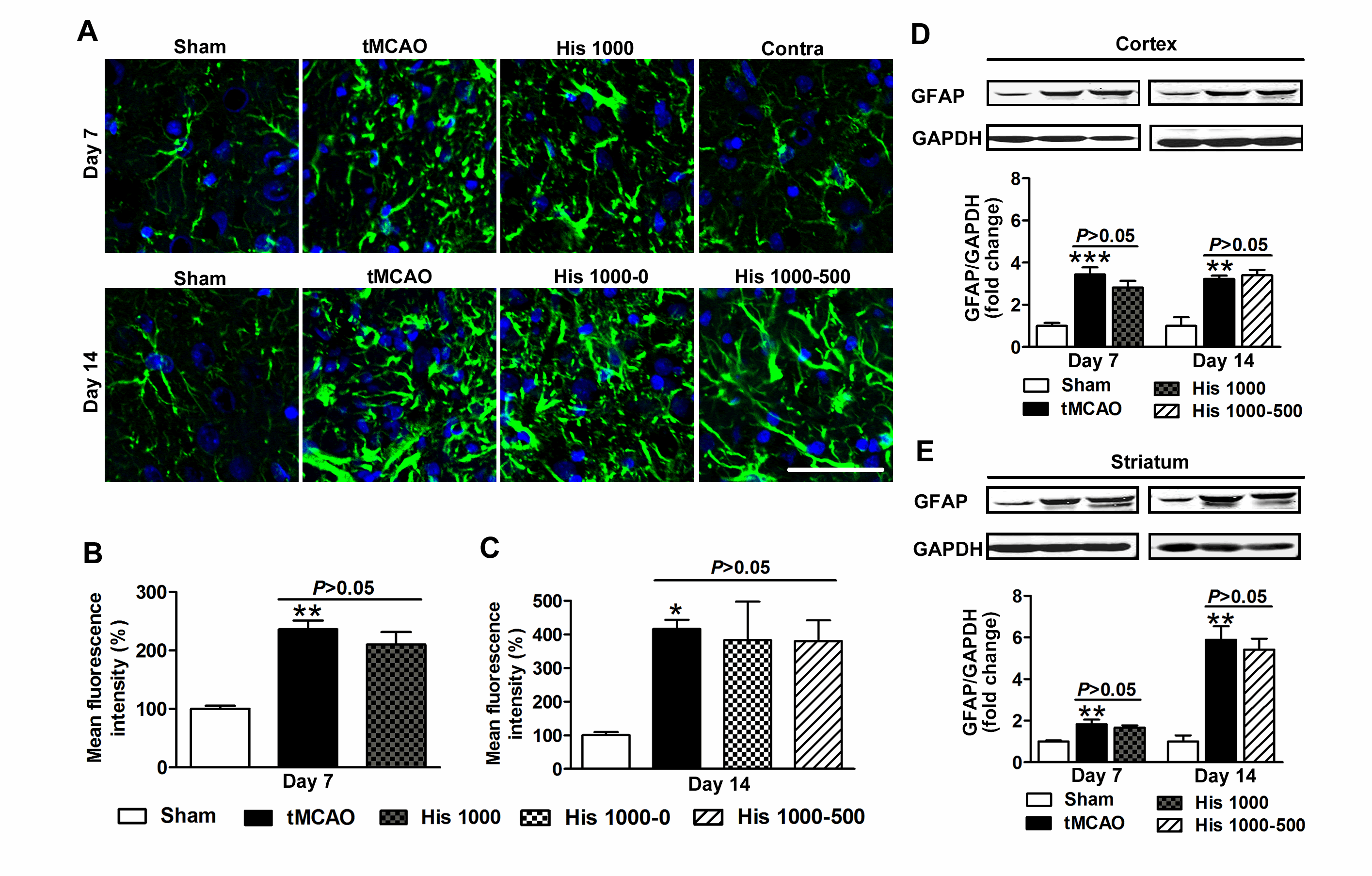
**

**Supplement Fig.2 Histidine treatment has no effect on the activation of astrocytes in the penumbra area after tMCAO.** GFAP immunostaining in the penumbra area or in the contralateral area (Contra) on 7 d and 14 d after tMCAO was shown in A (GFAP: green; DAPI: blue). The GFAP fluorescence intensity on 7 d (B) and 14 d (C) after tMCAO was quantified by Image J software. The GFAP expression in the cortex (D) and striatum (E) was also analyzed by Western blot. n=6-7. Bar=50 µm. **P*<0.05, ***P*<0.01, ****P*<0.001, compared with sham group.


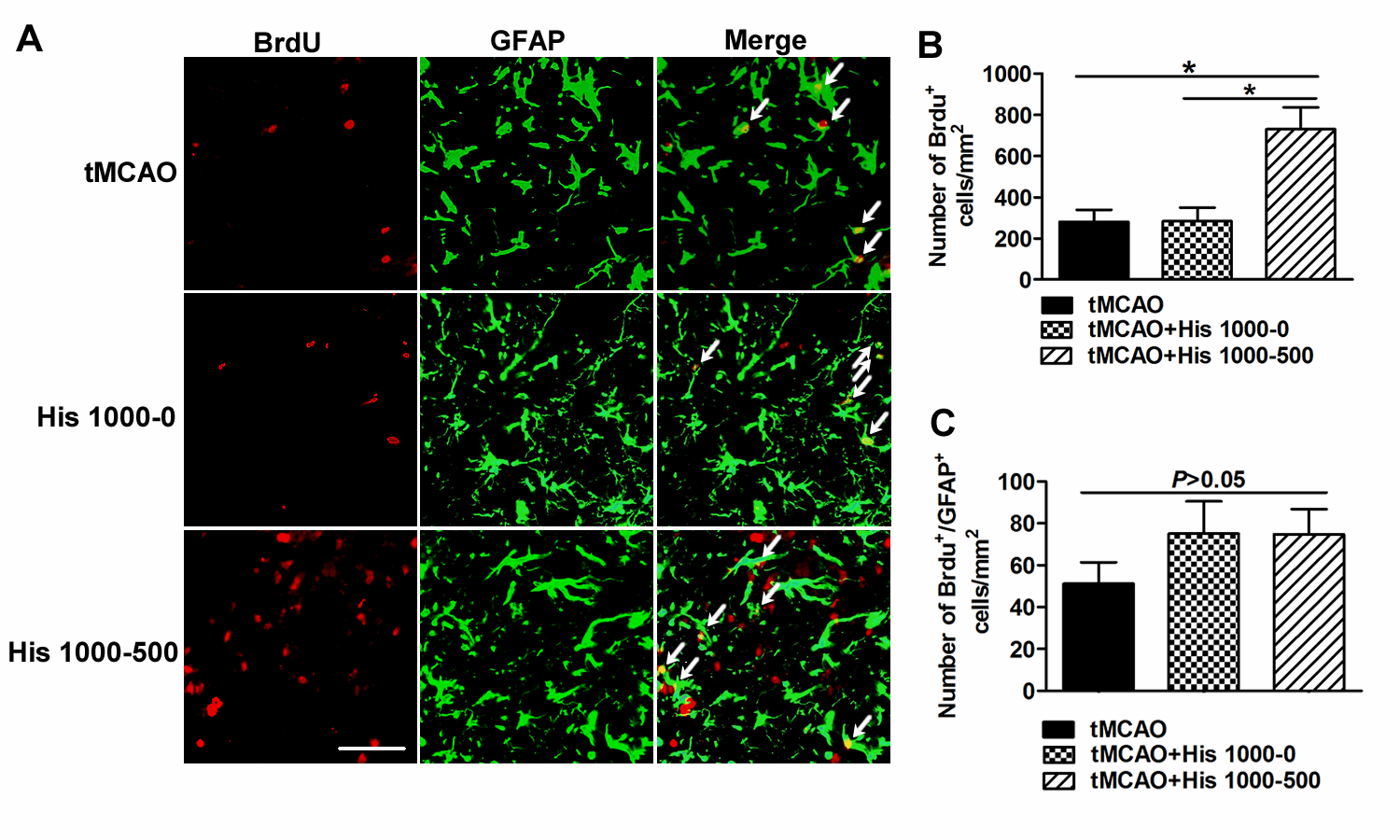


**Supplement Fig.3 Histidine treatment has no effect on the proliferation of astrocytes in the penumbra area after tMCAO**. Rats were received intraperitoneal injections of BrdU at 50 mg/kg twice daily at 0-14 d after tMCAO. On 14 d after tMCAO, co-immunostaining of GFAP and BrdU was performed. The sections were pretreated with 2 N HCl for 30 min. After repeated wash, sections were then incubated with 3% normal donkey serum in PBS containing 0.3% Triton X-100 for 15 min. Then primary antibodies for GFAP (1:400, boster, China) and 5'-bromo-20-deoxyuridine (BrdU; 1:1000; Sigma, USA) were applied for overnight incubation at 4 °C. After repeated wash in PBS, Alexa 488 conjugated anti-rabbit IgG (1:400; Invitrogen, CA) or Alexa 594 conjugated anti-mouse IgG (1:400; Invitrogen, CA) were applied for 2 h incubation at room temperature. The proliferating astrocytes in penumbra area were shown in A (GFAP: green; Brdu: red, with arrows). The number of BrdU^+^ cells (B) and BrdU^+^/GFAP^+^ cells (C) in the penumbra area was counted. n=6-7; Bar=50 µm. **P*<0.05.

**
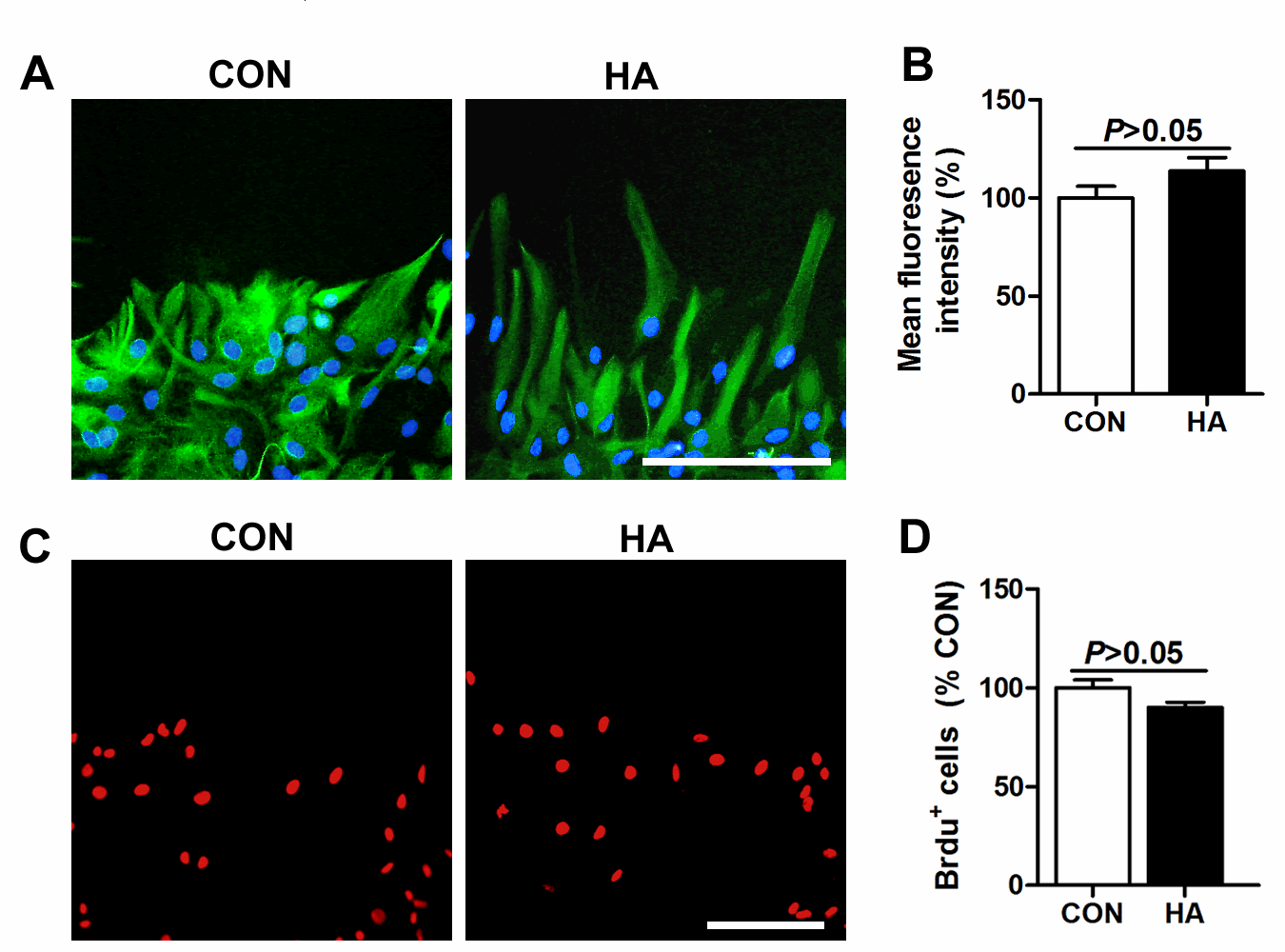
**

**Supplement Fig.4 Histamine has no effect on the activation and proliferation of astrocytes in wound-healing assay.** For BrdU incorporation analysis to assess proliferation, 10 μmol/L BrdU and 10^-7^ mol/L histamine (HA) were applied after scratch. GFAP or BrdU immunostaining was performed at 24 h after scratch. Briefly, the cells were fixed by 4% paraformaldehyde 15 min and treated with 2 N HCl for 30 min. After repeated wash, sections were then incubated with 3% normal donkey serum in PBS containing 0.1% Triton X-100 for 15 min. Then, cells were incubated with primary antibodies for GFAP (1:200, boster, China) or BrdU (1:1000; Sigma, USA) at 4 °C overnight. After repeated wash in PBS, Alexa 488 conjugated anti-rabbit IgG (1:400; Invitrogen, CA) or Alexa 594 conjugated anti-mouse IgG (1:400; Invitrogen, CA) were applied for 2 h incubation at room temperature. The GFAP immunostaining of astrocytes at the wound boundary area was shown in A (GFAP: green; DAPI: blue), and the fluorescence intensity was quantified (B). The number of BrdU positive cells at the wound boundary area was counted (D) with representative photographs in C. Values are from 3 to 4 independent experiments. Bar=250 μm.


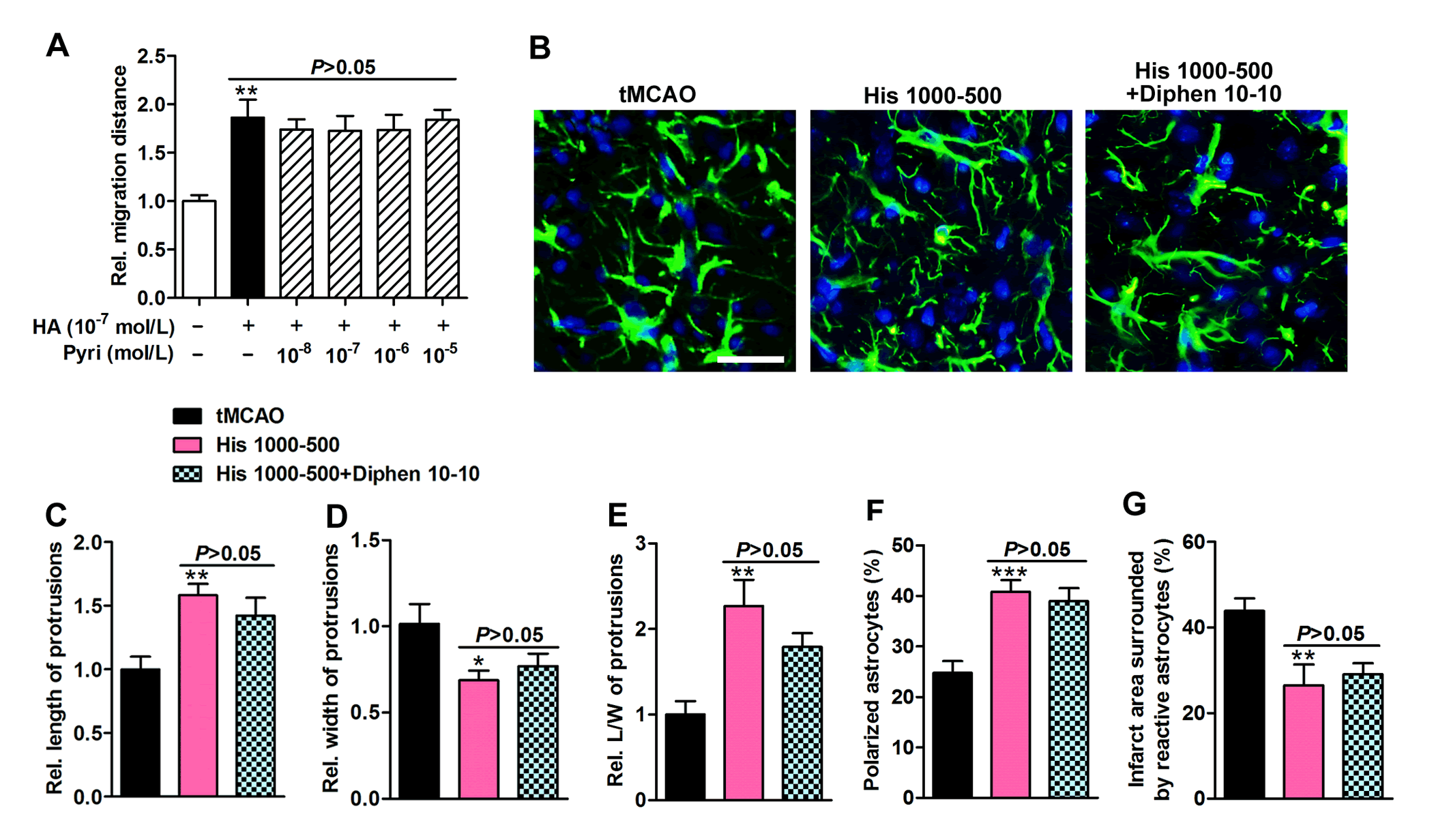


**Supplement Fig.5 H1 receptor is not involved in the promotion of astrocyte migration provided by histamine.** In wound-healing assay, pyrilamine (Pyri) at indicated concentration was administrated after the scratch, while 10^-7^ mol/L histamine (HA) was added 30 min later. Their effects on astrocyte migration at 24 h after scratch were shown in A. After tMCAO, diphendyadramine at 10 mg/kg (Diphen 10-10) was injected intraperitoneally 30 min before each injection of histidine. The morphology of astrocytes from the glial scar edge on 14 d after tMCAO was shown in B (GFAP: green; DAPI: blue). The length (C), the width (D), the ratio of length to width of protrusions (E) and the percentage of polarized astrocyte (F) were quantified. The infarct area surrounded by reactive astrocytes was measured (G). A: values are from 3 to 4 independent experiments; B-G: n=6-7; bar=50 μm. **P*<0.05, ***P*<0.01, ****P*<0.001, compared with control group (A) or tMCAO group (B-G).


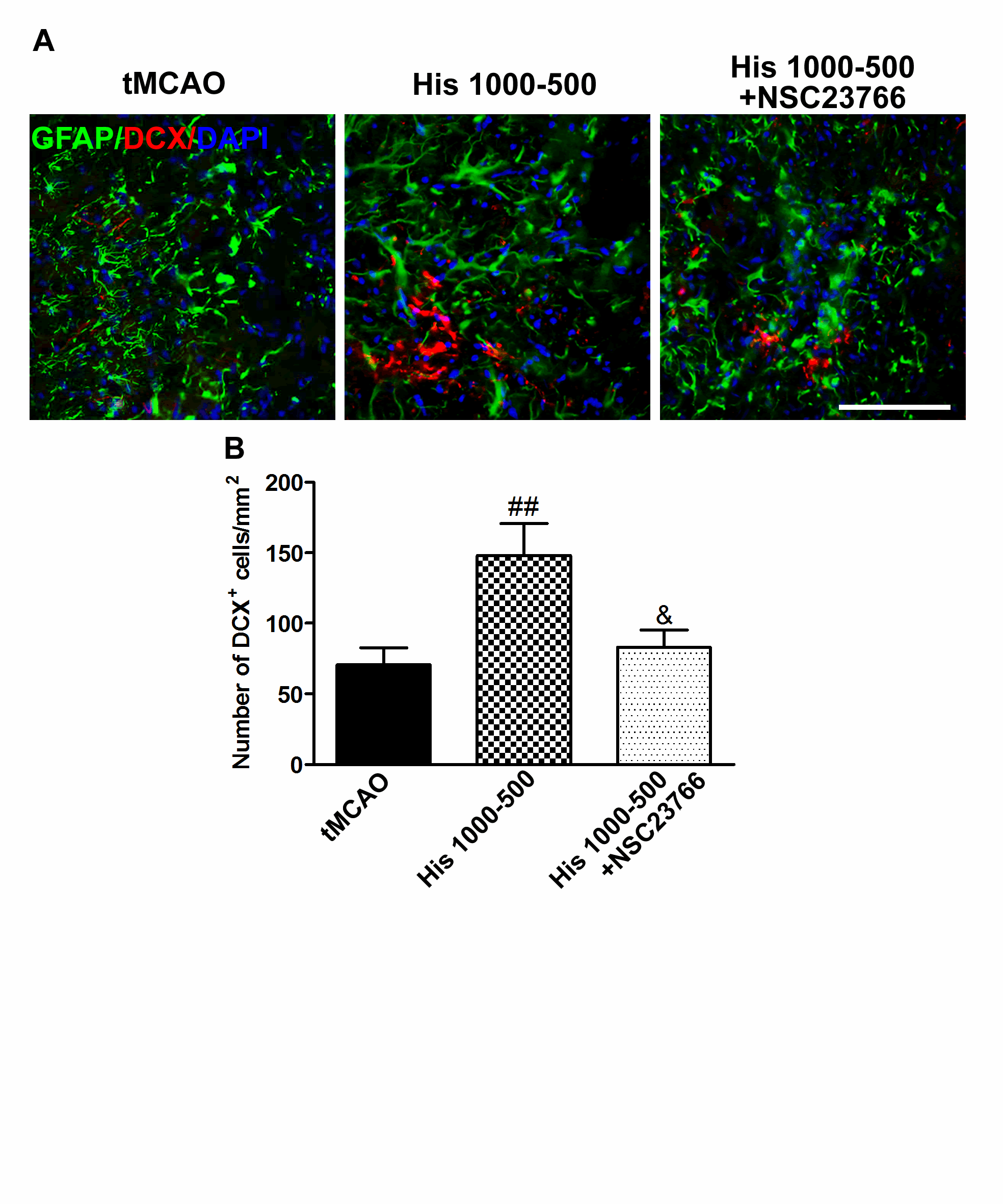


**Supplement Fig.6 Inhibition of small GTPase rac1 impedes the neurogenesis.** After tMCAO, 50 μg NSC23766 was delivered into cerebral ventricle at 30 min before each injection of histidine from 7 d to 14 d after tMCAO. On 14 d after tMCAO, co-immunostaining of GFAP and DCX (doublecortin) was performed by using anti-GFAP (1:400, Abcam, China) and anti-DCX (1:400; CST, USA). The DCX positive immature neurons at the glial scar edge were counted (GFAP: green; DCX: red; DAPI: blue). n=7; bar=100 μm. ##*P*<0.01, compared with tMCAO group; &*P*<0.05, compared with His 1000-500 group.


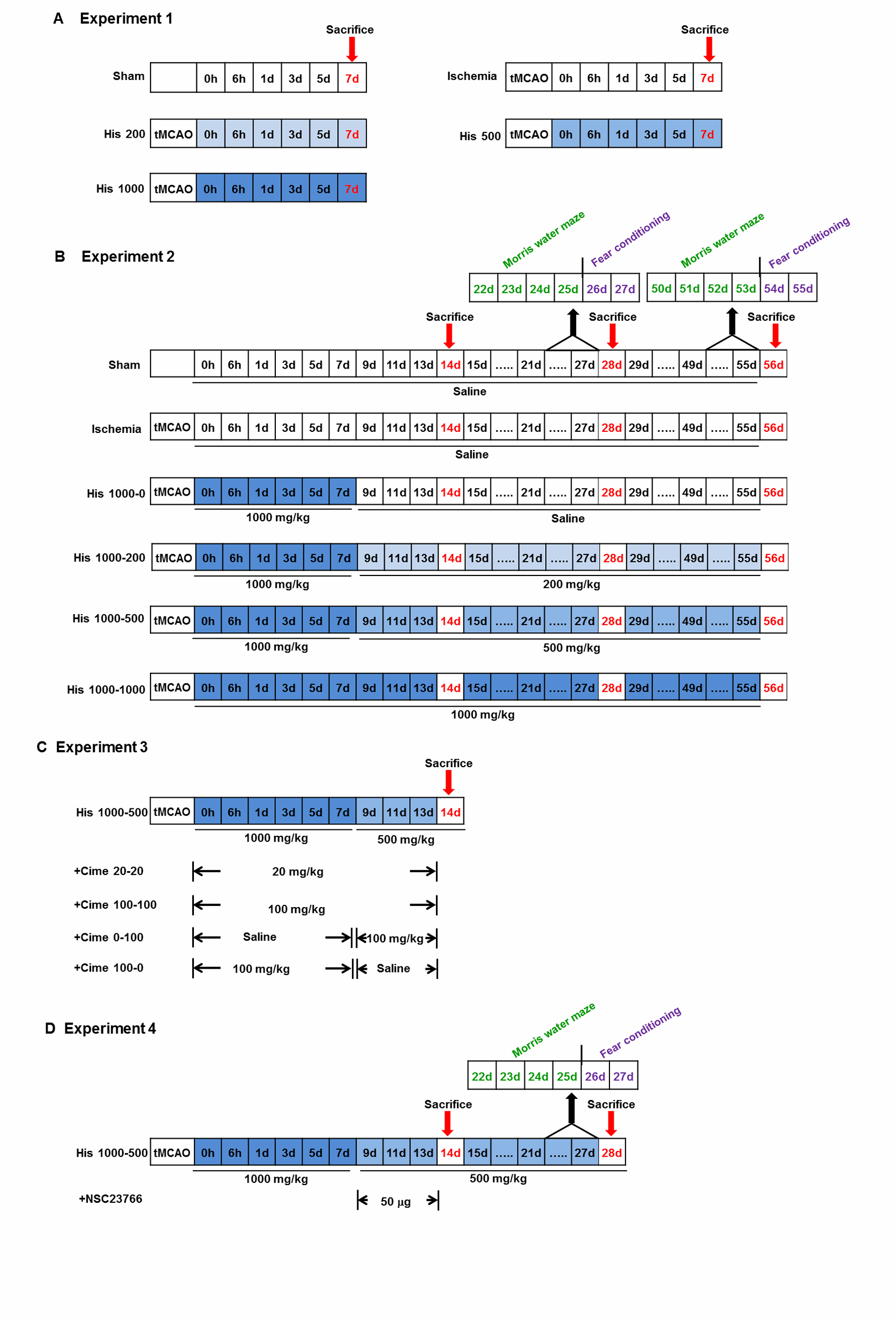


**Supplement Fig.7 Schematic diagram for the experiment schedule.** Experiment 1, rats were given 200, 500 or 1000 mg/kg histidine during the first week. Experiment 2, rats were given 1000 mg/kg histidine during the first week but 0, 200, 500 or 1000 mg/kg histidine during later weeks, which are named as His 1000-0, His 1000-200, His 1000-500 or His 1000-1000 groups. For histidine treatment, histidine was injected intraperitoneally at 0 h, 6 h and every other day after tMCAO. For the controls, rats were injected with the same volume of saline. Experiment 3, along with His 1000-500 treatment, cimetidine treatments were also divided into two phases (first week and later week), during which cimetidine (20 or 100 mg/kg) was given intraperitoneally 30 min before each injection of histidine, including Cime 20-20, Cime 100-100, Cime 0-100 and Cime 100-0 regimens. Experiment 4, along with His 1000-500 treatment, Rac1 inhibitor NSC 23766 (50 μg) was delivered to the contralateral ventricle 30 min before each injection of histidine during the second week after tMCAO. The neurological function were scored at 1, 3, 7, 14, 28, 42, 56 days after tMCAO and cognitive abilities in Morris water maze (Day 22-25, Day 50-53) and contextual fear conditioning (Day 26-27, Day 54-55) were evaluated before sacrifice. Rats were sacrificed at 7, 14, 28 or 56 days after tMCAO for immunohistochemistry, Western blot or TB staining.

**Supplement Table 1 Inclusion and exclusion animal number for ischemia experiments**

| Sacrificed time | Group | Included rats | | Death | | Exclusion^a^ | | Including Figures |
| --- | --- | --- | --- | --- | --- | --- | --- | --- |
| Day 7 | Sham | | 20 | | 0 | | 0 | Fig.1A;  Fig.3 A (Day 7), B (Day 7)  Supplement Fig.1; Supplement Fig.2A, B, D(Day 7), E (Day 7) |
|  | tMCAO | | 22 | | 6 | | 2 |  |
|  | His 200 | | 14 | | 4 | | 2 |  |
|  | His 500 | | 14 | | 4 | | 3 |  |
|  | His 1000 | | 22 | | 6 | | 2 |  |
| Day14 | Sham | | 6 | | 0 | | 0 | Fig.3A (Day 14), B (Day 14), C-G;  Fig.5;  Fig.6C-H;  Supplement Fig.2A,C,D (Day 14), E (Day 14)  Supplement Fig.3; Supplement Fig.5 |
|  | tMCAO | | 40 | | 12 | | 7 |  |
|  | His 1000-0 | | 7 | | 2 | | 1 |  |
|  | His 1000-500 | | 42 | | 12 | | 5 |  |
|  | His 1000-500  +Cime 20-20 | | 12 | | 3 | | 0 |  |
|  | His 1000-500  +Cime 100-100 | | 12 | | 4 | | 0 |  |
|  | His 1000-500  +Cime 0-100 | | 11 | | 3 | | 1 |  |
|  | 1000-500  +Cime 100-0 | | 12 | | 3 | | 0 |  |
|  | His 1000-500  +Diphen 10-10 | | 7 | | 2 | | 0 |  |
|  | His 1000-500  +NSC72366 | | 11 | | 4 | | 0 |  |
| Day 28 | Sham | | 23 | | 0 | | 0 | Fig.1B-E;  Fig.2A-D;  Fig.6 I-M |
|  | tMCAO | | 23 | | 9 | | 3 |  |
|  | His 1000-0 | | 14 | | 4 | | 2 |  |
|  | His 1000-200 | | 15 | | 4 | | 1 |  |
|  | His 1000-500 | | 25 | | 7 | | 3 |  |
|  | His 1000-1000 | | 15 | | 4 | | 1 |  |
|  | His 1000-500  +NSC72366 | | 12 | | 4 | | 0 |  |
| Day 56 | Sham | | 8 | | 0 | | 0 | Fig.1F, G;  Fig.2A, B, E, F |
|  | tMCAO | | 7 | | 2 | | 1 |  |
|  | His 1000-0 | | 7 | | 2 | | 1 |  |
|  | His 1000-200 | | 8 | | 2 | | 0 |  |
|  | His 1000-500 | | 7 | | 2 | | 1 |  |
|  | His 1000-1000 | | 9 | | 3 | | 1 |  |

^a^Number of rats excluded due to unsuccessful occlusion or reperfusion
